# Clinicopathological features of patients with wide local excision of eyelid malignant neoplasms: a more than five years retrospective study from China

**DOI:** 10.1186/s12886-022-02645-y

**Published:** 2022-11-15

**Authors:** Yuanyuan Han, Miao Kong, Yan Luo, Bin Sun, Zhiqiang Wang, Hong Zhang

**Affiliations:** 1 Department of Ophthalmology, Tianjin Medical University, No. 22, Qixiangtai Road, Tianjin, 300052 China; 2 Department of Orbit, Shanxi Eye Hospital, N0. 100, Fuxi Street, Shanxi, 030001 China; 3 Department of Strabismus, Shanxi Eye Hospital, N0. 100, Fuxi Street, Shanxi, 030001 China; 4 Department of Ophthalmology, Sino-Singapore Eco-City Hospital of Tianjin Medical University, No.3333, Hechang Road, Zhongxin Eco-City, Binhai New Area, Tianjin, 3000467 China

**Keywords:** Wide local excision, Eyelid malignant neoplasms, Clinicopathological feature, Recurrence, Mortality, Prognosis

## Abstract

**Background:**

To investigate the correlation between the clinical and pathological characteristics and outcomes in patients with eyelid malignant tumors underwent wide local excision.

**Methods:**

This retrospective study included 141 cases of eyelid malignant neoplasms from January 2010 to December 2015 in Shanxi eye hospital. Demographic and clinical information were collected. The Kaplan–Meier method was used to calculate survival curves, and the log-rank test method was used to compare survival between groups. Cox proportional regression models were used to calculate the hazard ratios (HR) of total recurrence rate and metastasis rate.

**Results:**

Overall, there were 141 eyelid malignant neoplasms cases aged 65.34 ± 9.69 (range, 41—88) years old. The duration time range was from 1 to 828 (61.09 ± 122.21) months. Basal cell carcinoma (BCC) is the most common of all eyelid malignancies, accounting for 84 (59.5%), followed by Sebaceous gland carcinoma (SGC, 41, 29%), Squamous Cell Carcinoma (SCC, 11, 7.8%), Malignant Melanoma (MM, 3, 2.1%)。On cox-regression analysis, pathological classification (HR 1.959; 95% CI 1.012–3.790; *p* = 0.046) and eyelid tumor surgery history (HR 17.168; 95% CI 1.889–156.011; *p* = 0.012) were independently associated with recurrence in patients with eyelid malignant neoplasm. Pathological classification (HR 2.177; 95% CI 1.423 -3.331; *p* < 0.001) was independently associated with metastasis in patients with eyelid malignant neoplasm. Recurrence and metastasis were most likely to occur in 3 years after surgery.

**Conclusion:**

Wide local excision is an effective and economical treatment for eyelid malignant neoplasms. The prognosis is mainly related to pathological types, eyelid tumor surgical history and TNM stages.

**Supplementary Information:**

The online version contains supplementary material available at 10.1186/s12886-022-02645-y.

## Introduction

Eyelid skin is not only the thinnest skin of the body but also the most common sunlight-exposed areas of skins, apart from subcutaneous fat layer, eyelid contains all other skin structures that can be the origin of various malignant tumors [1]. Eyelid malignant tumors were accounting for more than 20% of all eyelid tumors [2, 3]. Malignant eyelid neoplasms often required wide excision to ensure complete excision of the tumor. Mohs micrographic surgery (MMS) is the gold standard for treating a variety of cutaneous conditions. MMS is reliable in ensuring complete tumor excision and conserving the maximum amount of healthy tissue, and its disadvantages include the length of the procedure, the need for special equipment and the relatively high cost. Presently, the availability of the Mohs procedure is limited even in the UK [4]. In most parts of China, wide local excision is still the first -line therapy for eyelid neoplasms. There are differences in the constituent ratio and prognosis of eyelid malignant tumors among different races. There are relatively few reports on the overall prognosis of eyelid malignant tumors from China. Therefore, it is necessary to explore the prognosis of eyelid malignant neoplasms which underwent wide local excision therapy in China.

Herein, the aim of current study is to investigate the long-term correlation between the clinical and pathological characteristics and outcomes of patients with eyelid malignant neoplasms who received wide local excision.

## Methods

This retrospective, clinical based, single-center study adhered to the tenets of the Declaration of Helsinki, and was approved by the institutional review board (IRB) and ethics committee of Shanxi Eye Hospital, the number of the ethics committee document is SXYYLL-20211209. Written informed consent was waived for this study given its retrospective design.

### Patient population

This retrospective study was performed in Shanxi Eye Hospital. We reviewed the medical records of 141 continuously cases with histologically confirmed eyelid malignant neoplasms from January 2010 to December 2015. The eyelid malignant tumor classification was according to the AFIP (Armed Forces Institute of Pathology, AFIP) Atlas of Tumor Pathology: Tumors of the Eye and Ocular Adnexa [5]. Moreover, the stage of each case was graded according to initial clinical presentation using the eighth edition of the AJCC staging system (AJCC 8) for eyelid carcinoma [6].

Patients with clinical, radiological or laboratory evidence of metastasis at initial visit and without histopathology results were excluded from the study.

The clinical and pathological information of these patients included age, gender, duration time, surgical history, anatomical location, laterality, TNM staging (tumor, nodes, metastasis-classification, TNM), recurrence, metastatic and disease related mortality. The time of follow-up, tumor recurrence times and rates, and systemic status of the patients were recorded. All the information was organized in the data files by two full-time follow-up personnel to ensure the accuracy of data. And detailed structured phone interviews were performed for all cases. The tumor follow-up table is mentioned in attachment 1.

### Surgical treatment

In patients with clinically malignant eyelid neoplasms diagnosis, the lesion was biopsied locally, and after the lesion was shown histopathologically to a malignant eyelid tumor, a wide local excision was performed. The macroscopic safety margins must be at least 3 mm laterally for BCC or 5 mm in cases of sclerosing Basal cell carcinoma (BCC) or the cases whose tumor size is larger than 2 cm. The resection range of squamous Cell Carcinoma (SCC), sebaceous gland carcinoma (SGC) and malignant Melanoma (MM) was performed at least 5–6 mm around the clinical edge of the tumor. Skin flaps was performed to repair the eyelid defect if the operative margin was confirmed negative by the intraoperative frozen section. Reconstruction was performed according to different eyelid defects. Total upper lid reconstruction was performed with a Cutler-Beard flap. Cutler-Beard bridge flap is designed from the opposing lower eyelid. The repair of full-thickness defect of lower eyelid is divided into two layers, Hughes tarsoconjunctival flap for posterior lamellar reconstruction, the anterior lamellar was reconstructed with a skin-muscle transposition flap or Blasius flap. V–Y glabellar rotation was used to reconstruct the medial-canthal defect. The defect of lateral canthus was treated with Byron-Smith flap. If the defect is too large to be repaired with pedicled skin flap, we will repair it with free skin graft from the inner side of the upper arm. Exenteration is performed for the management of eyelid malignant tumors with intraorbital extension. During the orbital exenteration surgery, we tried to keep orbital bone unless a tumor invasion of orbital wall, because the orbital bone was a natural barrier against tumor invasion. The sectioning procedure was carried out by one trained pathology technician. All cases were confirmed by intraoperative frozen section and paraffin section after operation, and no tumor cells existed at the resection margin. All operations were performed by one surgeon team.

### Statistical analysis

Statistical analysis was performed using IBM SPSS Statistics for Windows, V.23.0 (IBM, Armonk, New York, USA). Categorical variables were presented as frequencies and percentages. Kolmogorov–Smirnov test was used to determine the normal of quantitative data. Continuous variables were descripted as mean and standard deviation (SD) or median (range) according to the description. Nonparametric Mann–Whitney U-test was performed to detect group differences for continuous variables, and χ [2] test or Fisher’s exact test was used for categorical variables. Continuous normality data were analyzed by one-way analysis of variance (ANOVA) and followed by post hoc analysis with the least significant difference (LSD) test. Kaplan–Meier method was used to draw the survival curve, and log-rank test was used to test the difference of survival curve between groups. Univariable and multivariable Cox proportional regression models were used to calculate the hazard ratios (HR) of total recurrence rate and metastasis rate, the confidence interval was 95%. All reported *p* values were two-sided. A *P* value of < 0.05 was considered as statistically significant.

## Results

**Fig. 1:**
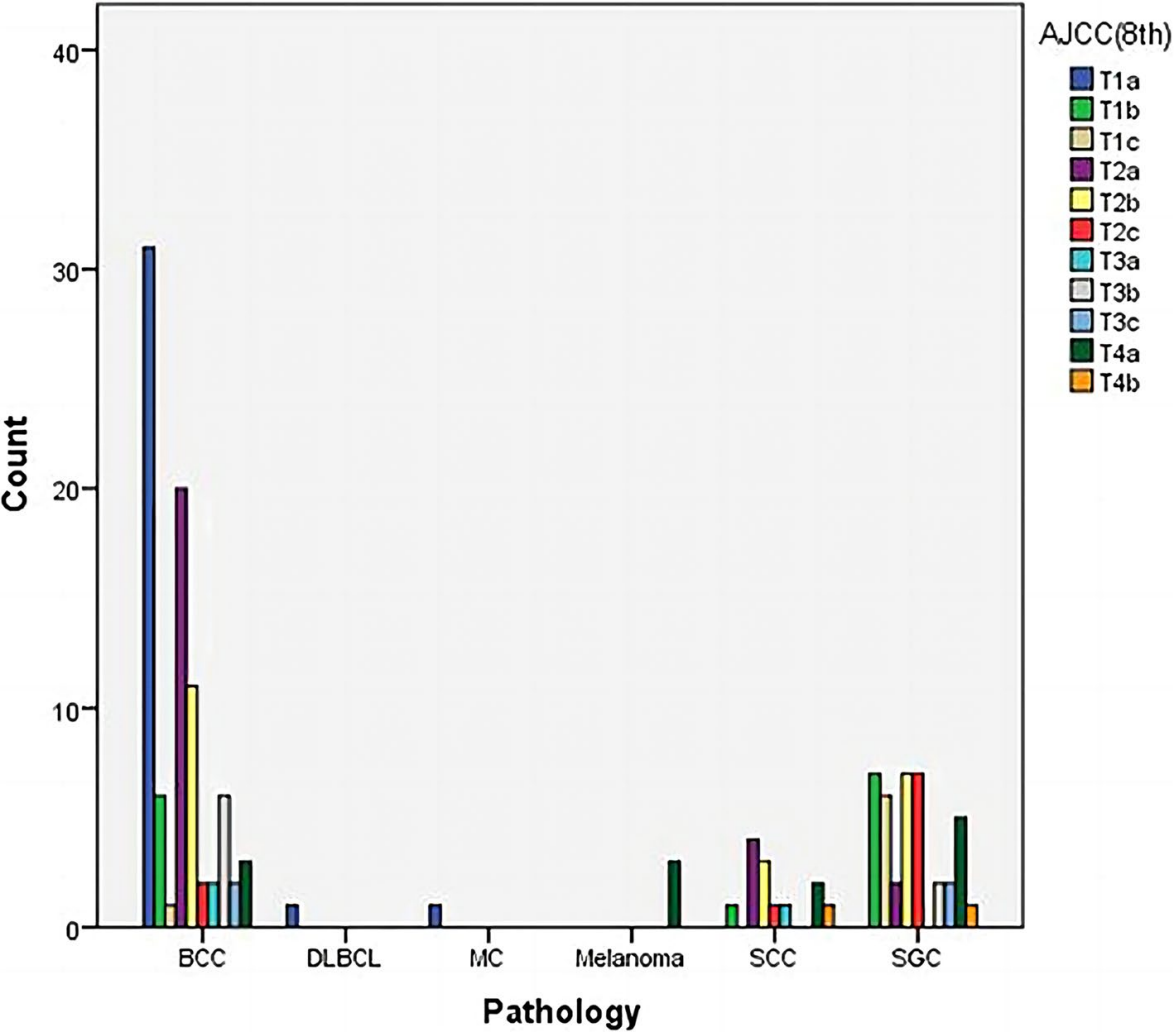
T stages for our patients with eyelid malignant tumors according to the 8th AJCC staging system

This study enrolled 141 patients composed of 65 (46.0%) males and 76 (54.0%) females in six years. Two cases of Basal cell carcinoma (BCC) occurred bilaterally. Demographic and clinical data were shown in Table 1. BCC is the most common type of all eyelid malignancies, accounting for 84 (59.5%), followed by Sebaceous gland carcinoma (SGC) with 41 cases (29%), Squamous Cell Carcinoma (SCC) with 11 cases (7.8%), Malignant Melanoma (MM) with 3 cases (2.1%), non-Hodgkin’s diffuse large B-cell lymphoma (1, 0.7%) and Mucinous carcinoma (1, 0.7%), respectively. The mean age at diagnosis was 65.34 years old (range, 41 – 88 years), and there is no significant difference in age between different pathological groups (*P* = 0.494). The duration time from onset of clinical symptoms to treatment ranges from 1 to 828 months with a mean of 61.09 $\pm$ 122.21 months. Among all types, patients with BCC have the longest duration time of disease with a mean of 88.83 $\pm$ 151.84 months (*P* < 0.001). For SGC, the delay from the time of onset of clinical symptoms until time of referral ranged from 1 to 72 months, with a mean of 22.90 $\pm$ 21.13 months. The mean duration time of SCC is 53.90 $\pm$ 64.72 months, ranges from 1 to 192 months. Male patients were more likely to be with SCC between male percentages in SCC, BCC, and SGC (*P* < 0.001). Among the 141 patients, 117 patients had no eyelid tumor history. 24 patients (17.0%) had eyelid tumor surgery history and received excision without negative margin control as the initial therapy at other institutions before they came to us. SCC was more likely to be misdiagnosed as other benign neoplasms and underwent surgery without margin control (*P* < 0.001). Both BCC and SCC were found predominantly in the lower eyelid accounting for 65.5%, and 45.6%, respectively. SGC is more common in the upper eyelid (58.5%, *P* < 0.001). (Table 1). The tumor size of 81.7% in all SCC in our study was greater than or equal to T2b. MM in this group was both greater than T2b. T stages for our patients with eyelid malignant tumors were showed in Fig. 1.

**Table 1:** Clinicopathological characteristics of the study groups

Characteristic	BCC (*n* = 84)	SGC (*n* = 41)	SCC (*n* = 11)	Melanoma (*n* = 3)	Other types (*n* = 2)	Total (*n* = 141)	*P*-value
Patient characteristics							
Mean age (years)	65.27 (44–88)	66.15 (41–88)	66.27 (54–77)	67.67 (62–71)	63.5 (63–64)	65.34 (41–88)	0.494
Gender male (%)	45.2	41.4	63.6%	33.3%	100	46	0.825
Duration (months)	88.83 ± 151.84	22.90 ± 21.13	53.09 ± 64.72	11.67 ± 10.97	5.57 ± 7.78	61.09 ± 122.21	0.007
Surgical History (yes)	6 (7.14%)	13 (31.7%)	2 (18.2%)	2 (66.7%)	1 (50%)	24 (17.0%)	< 0.001
Anatomical location, n (%)							
Upper eyelid	10 (11.9%)	24 (58.5%)	3 (27.2%)	1 (33%)	2(100%)	40 (28.37%)	< 0.001
Lower eyelid	55 (65.5%)	14 (34.1%)	5 (45.6%)	1 (33%)	0	75 (53.19%)	0.007
Medial canthus	13 (9.2%)	1 (2.4%)	1 (9.1%)	0	0	15(10.64%)	0.390
Lateral canthus	4 (4.9%)	1 (2.4%)	1 (9.1%)	0	0	6 (4.26%)	0.926
Whole	2 (2.4%)	1 (2.4%)	1 (9.1%)	1 (33%)	0	5 (3.55%)	0.074
Laterality							
OD	42 (50%)	26 (63.4%)	3 (27.2%)	2 (66.6%)	1(50%)	74 (52.4%)	0.265
OS	40 (47.6%)	15 (36.6%)	8 (72.7%)	1 (33.3%)	1(50%)	65 (46%)	0.296
OU	2 (2.3%)	0 (0%)	0 (0%)	0 (0%)	0	2 (1.4%)	0.848

*OD* Right eye, *OS* Left eye, *OU* Both eyes, *BCC* Basal cell carcinoma, *SGC* Sebaceous gland carcinoma, *SCC* Squamous cell carcinoma

**Fig. 2:**
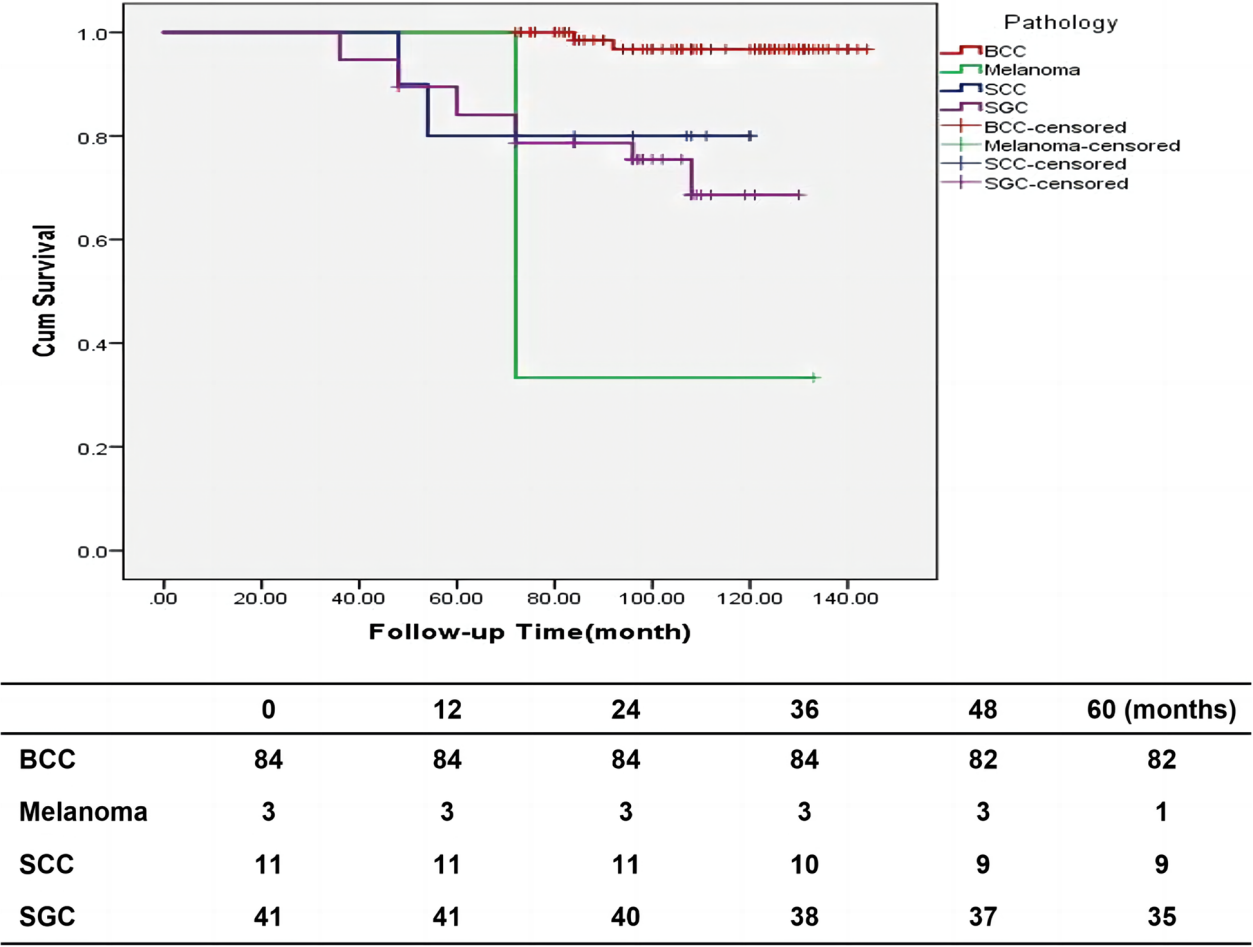
Cumulative survival for different pathological, statistically significant difference (P < 0.001) detected in different pathological. BCC, Basal cell carcinoma; SGC, Sebaceous gland carcinoma; SCC, Squamous cell carcinoma

**Fig. 3:**
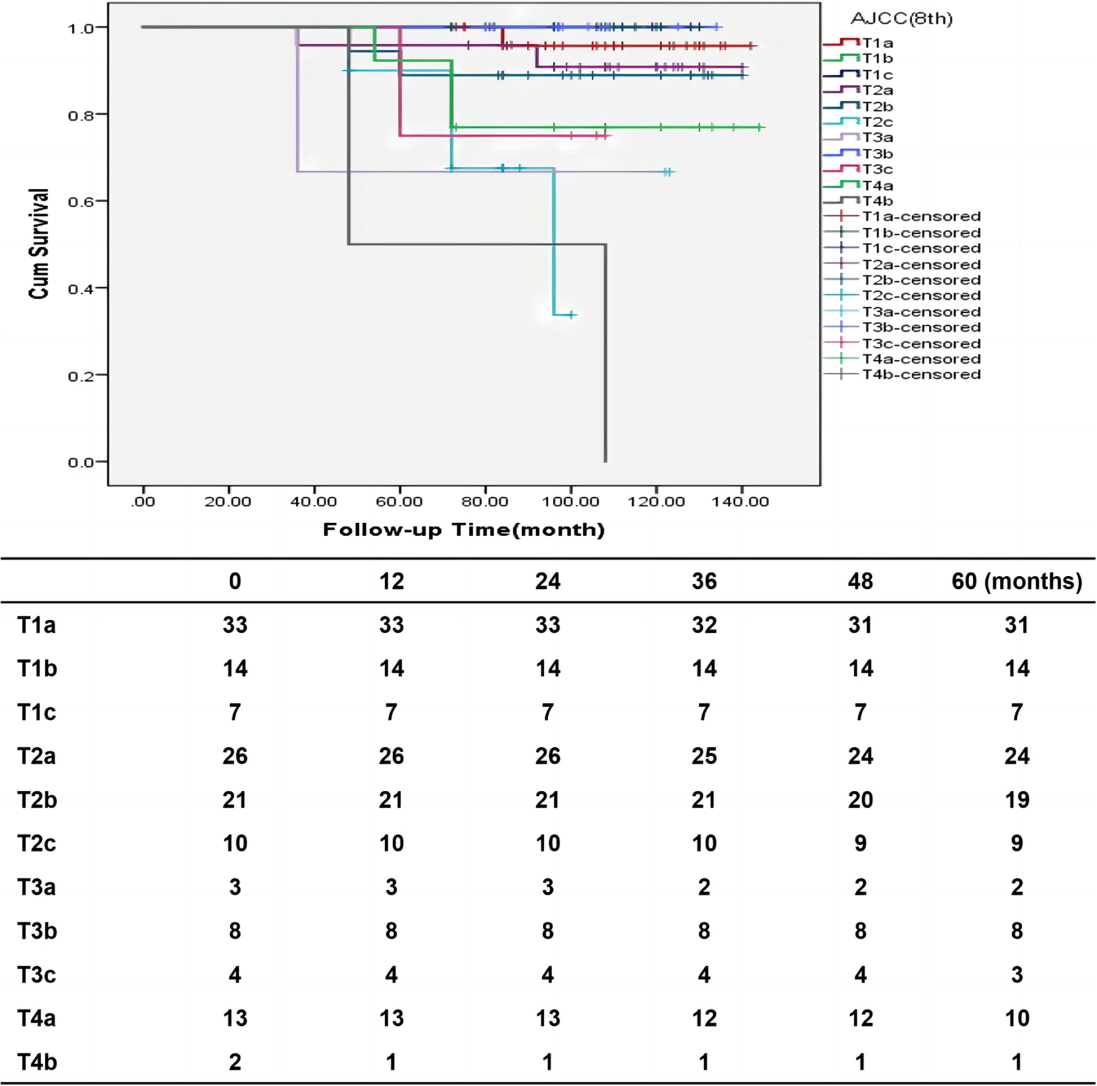
Cumulative survival for AJCC period, statistically significant difference (*P* = 0.007) detected in different AJCC period

**Fig. 4:**
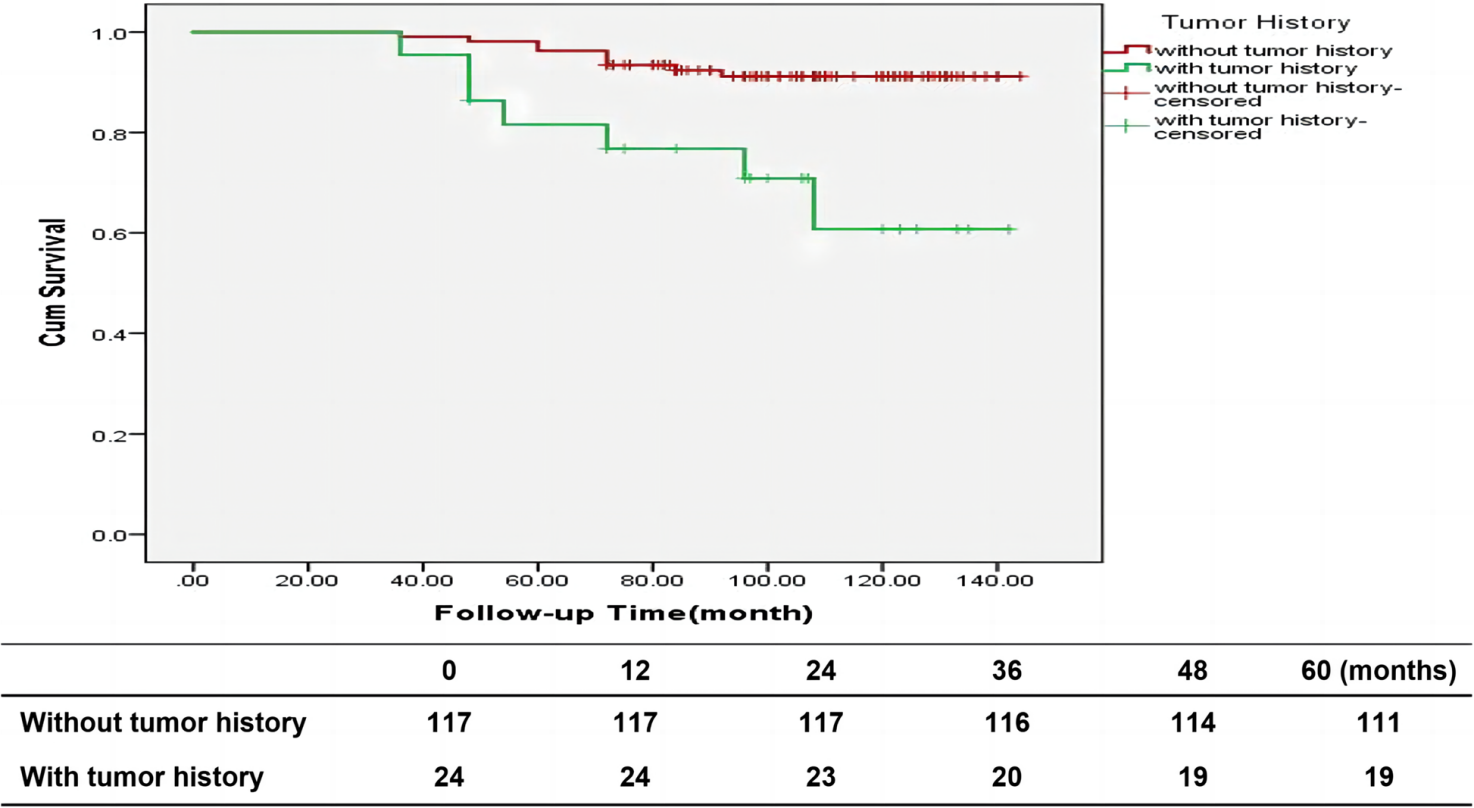
Differences in cumulative survival rates with and without surgical history, statistically significant difference (*P* = 0.006) detected in groups whether the patients underwent an eyelid tumor surgery history

One hundred and fourteen patients had been alive till December 2021 with mean follow-up time of 102.52 (36–144) months. 11 patients were lost of follow-up. Of which, 6 cases were BCC, one was MM, one was SCC and 3 cases were SGC. The loss ratio of follow-up was 7.8%. The mean overall survival was 61.09 $\pm$ 10.29 (72– 144) months. At the end of the follow-up period, the overall survival rate was 97.3% for BCC, 75.7% for SGC, 81.9% for SCC, and 33.3 for MM (Fig. 2). The 5-year survival rate of BCC patients undergoing wide local excision was 97.3%, and the eyelid tumor-related mortality rate was 0. Tumor-related mortality was 0 for BCC, 26.3% for SGC, 20% for SCC, 66.6% for MM, 100% for non-Hodgkin's lymphoma, and 0 for mucinous adenocarcinoma. There were 17 patients (10 patients had SGC, 2patients had SCC, 2 patients had BCC, 2 patients had Melanoma and the other one had non-Hodgkin’s diffuse large B-cell lymphoma) had died with median follow-up time 60 (36– 96) months. Two patients with BCC died of original lung squamous cell carcinoma. Tumor-related mortality were shown a significant difference between different pathological groups (*P* < 0.001). The five years tumor-related mortality was 0 in BCC group and 20% in SCC group, detailed information was shown in Table 2. Overall, we did find that higher T stage was significantly associated with tumor-related mortality (*P* < 0.001), Details information was shown in Fig. 3. The overall survival rate of patients with no surgical history of eyelid lesions was 93.1%, and that of patients with surgical history of eyelid tumors (without negative margin control) was 87%. Tumor-related mortality were shown a statistically difference between patients with or without eyelid tumor surgery history (*P* = 0.001), and the detailed information was shown in Fig. 4.

**Table 2:** Comparison of recurrence and mortality between four main types different pathological malignant eyelid tumors

Follow-up	BCC (*n* = 78)	SGC (*n* = 38)	SCC (*n* = 10)	Melanoma (*n* = 2)	*P*-Value
Mean follow-up time (months)	108.63 ± 2.37	94.97 ± 3.71	96.50 ± 7.21	92.33 ± 20.33	0.003
5-year recurrences	0 (0%)	4 (10.5%)	1 (10%)	2 (100%)	< 0.001
5-year tumor-related mortality	0 (0%)	10 (26.3%)	2 (20%)	0 (0%)	< 0.001

*BCC* Basal cell carcinoma, *SGC* Sebaceous gland carcinoma, *SCC* Squamous cell carcinoma

Seven cases recurred, the median time of recurrence was 3 (1 – 6) years. Of which, 4 cases were SGC, 2 cases were SCC and 1 case was Non-Hodgkin’s diffuse large B-cell lymphoma (DLBCL). The recurrence rate of SCC was 15.3% in our series. Six patients (85.7%) underwent an eyelid tumor surgery history without the clear excision margins before they came to us. The mean time to recurrence (recurrence-free time) was 34 months for SCC, the highest recurrence rate is observed within three years after the surgical act. On cox-regression analysis, pathology (HR 1.959; 95% CI 1.012–3.790; *p* = 0.046) and eyelid tumor surgery history (HR 17.168; 95% CI 1.889–156.011; *p* = 0.012) were independently associated with recurrence in patients with eyelid malignant neoplasm, detailed information was shown in Table 3. Metastasis occurred in 17 cases. On cox-regression analysis, pathological classification (HR 2.177; 95% CI 1.423 -3.331; *p* < 0.001) was independently associated with metastasis in patients with eyelid malignant neoplasm, detailed information was shown in Table 4.

**Table 3:** Independent risk factors according to recurrence by Cox regression analysis

Vairable	Univariate analysis		Multivariate analysis	
	HR (95%CI)	*P*-Value	HR (95%CI)	*P*-Value
Pathology	2.707 (1.583–4.630)	< 0.001	1.959 (1.012–3.790)	0.046
Eyelid tumor history	30.444 (3.658–253.390)	0.002	17.168 (1.889–156.011)	0.012
TNM staging	0.877 (0.393–1.956)	0.748		

*HR* Hazard ratio, *CI* Confidence interval, *TNM* staging (tumor, nodes, metastasis-classification)

**Table 4:** Independent risk factors according to metastasis by Cox regression analysis

Vairable	Univariate analysis		Multivariate analysis	
	HR (95%CI)	*P*-Value	HR (95%CI)	*P*-Value
Pathology	2.464 (1.738–3.494)	< 0.001	2.177 (1.423–3.331)	< 0.001
Eyelid tumor history	4.588 (1.758–11.973)	0.002	2.149 (0.738–6.255)	0.160
TNM staging	1.735 (1.127–2.671)	0.012	1.202 (0.781–1.848)	0.403

*HR* Hazard ratio, *CI* Confidence interval, *TNM* Staging (tumor, nodes, metastasis-classification)

Seven patients underwent orbital exenteration because of the orbital infiltration (4 cases of SGC, 2 cases of BCC and 1 case of melanoma). Unfortunately, the Patient with malignant melanoma died after 6 years from the orbital exenteration because of metastatic dissemination to the brain. Other patients were still alive at the end of the follow-up period.

## Discussion

According to our findings, BCC was found to be the most common type of eyelid malignancies. The prognosis of tumor is related to its pathological classification, and BCC has the best prognosis. BCC may be associated with a primary secondary tumor—lung squamous cell carcinoma. The disease composition of our study is similar to that of previous Asian populations [6–10].

The overall mean follow-up time after treatment was 102.52 months (36–144), the follow-up time is longer than most of the previous reports (between 2 months and 4 years) [11]. Because of the long follow-up time, our results are more likely to reflect the long-term outcomes of eyelid malignancies treated with wide local excision. Through our long-term follow-up of at least 5 years, the survival time of the tumors that received wide local excision was primarily related to the pathology of the eyelid tumors. The tumor-related mortality of BCC after wide local excision was significantly lower than that of MMS and other surgical procedures [12]. An unexpected finding in our study was that two patients with BCC were associated with a second tumor, original lung squamous cell carcinoma, which had not been reported in the previous literature. There were few reports on metastasis of basal cell carcinoma, The published incidence of metastasis is from 0.0028 to 0.55%. Thomas et al. reported a lung metastases in a case of basal cell carcinoma of the eyelid, Whether this tumor arises de novo or develops from an existing BCC is debated, Thomas supports the former scenario [13]. The two patients with lung squamous cell carcinoma were both 75 years old with no tumor history or family history. The tumor size was T2a and T2b, respectively. The anatomical location of one patient was on the upper eyelid and the other on the lower eyelid, but both were located on the temporal side. No local recurrence occurred in the both patients. The mechanism of original second lung squamous cell carcinoma in the two patients is unclear. Attention should be paid to the lung condition of patients with BCC, although the incidence is very low. Song et al. reported that the 3–5 years mortality rate of SGC is 25.6%, and they adopted the method of paraffin section to control the incision margin. If the incision margin is positive, it needs to be enlarged again, and then eyelid reconstruction is performed after the incision margin is clean [14]. This method increases the times of biopsies and the length of surgery. The use of wide local excision in SGC was verified to reliable through our study, and its mortality was greatly reduced compared with previous reports [15]. Mortality in SCC was 20%, significantly higher than previously reported (1.9–14.7%) [16]. We speculate that the higher recurrence rate of SCC in our study is associated with the high TNM staging. The tumor size of 81.7% in all SCC in our study was greater than or equal to T2b. The mortality of MM in our case was 66.6%, but their 5-year mortality was 0 because both patients died in the sixth year after surgery. Breslow et al. found that lesions measuring 0.76 mm or less were associated with a 5-year survival rate of 100%, whereas patients with tumors that had invaded more than 1.5 mm had a 5-year survival rate being 50% to 60% [17]. In our study, the maximum transverse diameters of both patients with MM were greater than 20 mm at the time of initial treatment. At the time of initial treatment, MM in this group was both greater than T2b, which may be the reason for the high mortality of this group of cases.

TNM stage was also a factor influencing overall survival rate. The overall survival rate of T4b stage was the lowest, followed by T2c stage. Joshua Ford et al. investigated the prognostic value of the 7th Edition of AJCC in eyelid malignant tumor and concluded nodal metastasis was significantly correlated with T2b or more extensive tumor at presentation [18]. Yun Hsia [6] et al. validate the performance of the T category of the 8th edition AJCC staging systems, they concluded tumors classified as T2c or worse had higher risk of regional lymph node metastasis, while tumors T3b or worse in the 8th edition had more tumor-related death [6]. In general, we should pay more attention to T2c or worse.

The recurrence of BCC is very low, and Poignet el al. concluded that after complete resection of BCC with negative margins, the rate of local recurrence is less than 1% at 5 years of follow-up; with incomplete excision, the local recurrence rate can be as high as 38% at 5 years [12]. Our cohort showed no recurred case in the cases followed up. One patient developed BCC in the contralateral eye 7 years after surgery. The time of last follow-up, 4 SGC patients (10.53%) recurred and 1 of the 4 “recurrences” developed pagetoid recurrence at new sites that were previously not involved by tumor, this result is similar to previous reports [19]. our study had a longer follow-up period. The advantage of our study was that the mean follow-up time of SGC was 94.97 $\pm 3.71$(36–132) months, compared with 20 to 44 months in previous studies. In this group of cases, two malignant melanomas recurred 3 years after surgery, the recurrence rate was 100%. Such a high recurrence rate was also associated with the TNM stage greater than or equal to T3a at the time of MM visit. Two more rare neoplasms were non-Hodgkin's lymphoma and mucinous adenocarcinoma. Non-Hodgkin’s lymphoma recurred locally 3 years after surgery, and mucinous adenocarcinoma was free of recurrence at the end of the follow-up period. Previous reports have suggested that the mean time to recurrence was 20 months and most carcinomas recurred within 2 years of surgery [15, 20]. Our study believed that the recurrence time of eyelid malignant tumor was 3 years after surgery, so the follow-up frequency within 4 years after surgery should be once every six months and switch to once every year after 4 years postoperative. We performed multivariate Cox regression on pathological classification, surgical history of the eyelid tumor and TNM stage. The results showed that the pathological classification and surgical history of the eyelid tumor was an independent risk factor for relapses. Patients who had a surgical history of eyelid tumor had a higher recurrence rate even after extensive resection with negative margin. The reason for eyelid tumor resection without margin control is that malignant eyelid tumor was misdiagnosed as benign eyelid tumor and underwent surgery without margin control at that time. Many ophthalmologists, however, are not intimately familiar with the clinical manifestations of malignant neoplasms of the eyelids, which are reasons for misdiagnosis. It is necessary for eye tumor specialists to carry out continuous education on eyelid malignant tumor to general ophthalmologists, so as to reduce the misdiagnosis rate of eyelid malignant tumor.

At the end of follow-up, 17 patients had metastases. Cox regression model showed that pathological classification was an independent risk factor for metastasis. Three cases of SGC died from liver metastasis, lung metastasis and parotid gland metastasis, respectively, without recurrence at the primary site of tumor. The time of discovery of metastasis was 5 years after surgery. Tumors larger than stage T2b at initial diagnosis were present in all deaths. Three SCC patients died at the end of follow-up, two died of pulmonary metastasis and one died of intestinal metastasis. The time of metastasis was 2 to 8 years postoperatively. All SCC deaths were larger than T2b and had local recurrence. Both MM patients died 6 years after surgery, one died of brain metastasis and the other died of lung metastasis. Previous reports have suggested that MM is more prevalent in liver metastases [21]. Paola et al. suggested that Patients affected by advanced malignant eyelid carcinoma need to be strictly controlled because metastasis can develop at least 5 years after surgical treatment [22].

Orbital exenteration surgery, a procedure that is catastrophic in terms of ocular function and appearance, was administered sparingly but only when eyelid neoplasms are potentially fatal or relentlessly progressive cannot be treated more effectively in other ways [23]. In our study, 7 patients underwent orbital exenteration, of these patients, 29% were BCC, 57% were SGC and 14% were malignant melanoma. The 5-year survival after surgery was 100%, one patient with MM died of brain metastatic neoplasm in 6 years after surgery. Although orbital exenteration is a severely disfiguring procedure, it is still an effective and economical treatment for eyelid malignancies that cannot be controlled by simple excision or radiotherapy [24].

In general, our study found that 4–5 mm incision edge for BCC, and 5–6 mm incision edge for SGC, SCC and MM were practicable, because both intraoperative frozen section and postoperative paraffin section confirmed that the resection edge of the tumor was controlled by the tumor resection edge. Wide local excision can reduce the times of eyelid tumor biopsy and shorten the operation time. There is no need for special pathologists to cooperate with ophthalmologist. It is more suitable for ophthalmic hospitals with a large number of patients and no professional pathologists to cooperate.

However, there were some limitations in our study. First, all patients were recruited from a single-center and hospital based design. Secondly, as a retrospective study, it’s also limited in terms of recall of patients and makes our findings skewed. Last but not least, the explanation for variations of eyelid malignant neoplasms is merely based on speculation, needs further mechanism investigation.

## Conclusion

Wide local excision is an effective and economical treatment for eyelid malignant neoplasms. Histologic type is the most important prognostic indicator for malignant eyelid neoplasms. BCC had the best prognosis, followed by SGC, SCC and MM. T4b stage has the lowest survival rate. The pathological classification and surgical history of the eyelid tumor was an independent risk factor for relapses. The pathological classification was an independent risk factor for metastasis.

## Supplementary Information

**Additional file 1.**

## Data Availability

The datasets used and analysed during the current study available from the corresponding author on reasonable request.
